# Evaluating functional outcomes in adolescents with attention-deficit/hyperactivity disorder: development and initial testing of a self-report instrument

**DOI:** 10.1186/s12955-015-0302-9

**Published:** 2015-08-22

**Authors:** Asha Hareendran, Juliana Setyawan, Robin Pokrzywinski, Anna Steenrod, Manisha Madhoo, M. Haim Erder

**Affiliations:** Outcomes Research, Evidera, London, UK; Global Health Economics Outcomes Research, Shire, Wayne, PA USA; Outcomes Research, Evidera, Bethesda, MD USA; Shire Global Medical Affairs, Shire, Wayne, PA USA; Global Health Economics and Epidemiology, Shire, Wayne, PA USA (Affiliation at the time of the study)

**Keywords:** ADHD, Adolescent, Cognitive interview, Concept elicitation, Content validity, Development, Functional outcome, Instrument, PRO

## Abstract

**Background:**

Engaging adolescents in decisions about their health may enhance their compliance with treatment and result in better health outcomes. Treatment outcomes in attention-deficit/hyperactivity disorder (ADHD) are rarely evaluated from the adolescents’ point of view. There is also concern that adolescents with ADHD may not have insight about the impacts of their disease. This article describes research conducted to understand the experiences of adolescents with ADHD and how the research was used to develop an adolescent self-report instrument.

**Methods:**

This research involved an iterative process to ensure content validity and was conducted in the following stages: concept identification from literature reviews and interviews with teachers and clinicians; concept elicitation interviews with adolescents with ADHD and their caregivers, review of existing instruments; development of a new instrument and cognitive interviews. Experts in instrument development and translation and clinical practitioners in ADHD also participated.

**Results:**

A conceptual framework to measure the impact of ADHD on adolescent functioning identified from concept identification research informed concept elicitation interviews with 60 adolescents with ADHD and their primary caregivers. In the interviews, adolescents discussed difficulties with performing activities in various contexts: school, home, leisure activities and social interactions. Caregivers provided additional insights. The instrument review revealed that none of the existing instruments were suitable to collect data on the elicited concepts; therefore, a new instrument was developed. Revisions were made to the format and content of the instrument (a daily diary) based on feedback received from cognitive testing with 15 adolescents.

**Conclusions:**

Our research helped to obtain a comprehensive understanding of the impacts of ADHD on adolescent functioning, to inform the development of a new instrument for measuring outcomes. Adolescents were able to discuss the impact of ADHD on their lives in concept elicitation interviews and report the impacts of ADHD on a self-report instrument. The new instrument developed to reflect the perspective of adolescents with ADHD can be used to supplement outcome assessments in clinic and research settings. Scientific advocacy for the use of such measures can be valuable to measure outcomes meaningful to adolescents with ADHD and the clinical community.

## Background

Treatments for attention-deficit/hyperactivity disorder (ADHD) aim to address symptomatic, syndromatic and functional outcomes of the disease [1, 2]. Medications for ADHD are effective in controlling symptoms such as inattention, overactivity and impulsivity [3, 4]. However, approximately 50–75 % of children with ADHD continue to suffer impacts associated with the disease into adolescence, despite receiving treatment [5]. Increased cognitive demands on adolescents to appropriately function in academic and social settings often result in greater morbidity owing to ADHD [6]. Treatment goals set for ADHD in childhood may change or expand as patients navigate the transitions to adolescence and early adulthood. For example, adolescents typically have longer school days and participate in more extracurricular and after-school social activities than younger children, so they may require treatment effects that last for a longer time during the day. Therefore, clinical practice guidelines [7, 8] for the treatment of ADHD encourage the collection of data from adolescents on concepts that reflect their experiences, to determine targets for treatment, and for monitoring and evaluating outcomes of interventions for the disease.

In addition to their changing environments, as children grow older they are given more responsibilities by their caregivers, and often make decisions about medication use. Expectations about outcomes of treatment may be different for adolescents with ADHD compared with those of younger children or adults with the condition. Adolescents’ beliefs about ADHD and attitudes to medication have also been shown to drive their adherence to treatment [1, 9, 10], which may improve if adolescents experience treatment benefits in the functional aspects most important to them. Additionally, the desire for more autonomy and control over their condition contributes to adolescents’ desire to make decisions about medication treatment [10].

However, the evaluation of treatments for ADHD in clinical trials have mainly used clinician or caregiver-reported assessments about the symptoms of ADHD, such as the ADHD-Rating Scale [11], and Connors’ Parent Rating Scale/Connors’ Teacher Rating Scale [12] to support clinical evaluations of benefit, as described in the approved product labels for ADHD medications. Larger discrepancies have been found in ratings of psychopathology between caregiver and teens than between caregivers and younger children. These larger discrepancies have been attributed to the lack of caregiver awareness of problematic behaviours and inconsistency in symptom manifestation across contexts (e.g., home, school) [13]. Parents often have less contact with adolescents compared with younger children, and may be less aware of adolescents’ day-to-day functioning. Interactions between adolescents and caregivers are also likely to change during adolescent years, with adolescents sharing less about their experiences [14]. Therefore, caregiver assessments may provide a different perspective to what is actually experienced by the adolescents, reaffirming the importance of collecting self-reported data. Collecting data about functional outcomes that are meaningful and easily understood by adolescents may contribute to better understanding of the full impact of ADHD, and for evaluating the benefits of treatments for ADHD. In addition, guidelines for evaluating medicinal products for the treatment of ADHD also highlight the importance of including adolescents’ perspectives to supplement clinicians’ assessments of symptoms [15].

The objective of this study was to identify impacts of ADHD that are most relevant to adolescents to help develop a strategy for evaluating treatment outcomes in this patient group. The study also aimed to explore the feasibility and options available for collecting adolescent self-reports that capture these impacts to monitor and evaluate outcomes of treatment.

The conduct of the study was guided by the methods described in the US Food and Drug Administration (FDA) Guidance for Industry Patient-Reported Outcome (PRO) Measures: Use in Medical Product Development to Support Labeling Claims [16] and the ISPOR Task Force Reports on best practice for establishing and reporting on content validity of a new PRO instrument [17]. The study involved an iterative process to ensure content validity, and was conducted in the following stages – *concept identification* from literature reviews and interviews with teachers and clinicians, *concept elicitation* interviews with adolescents with ADHD and their caregivers, development of conceptual frameworks, review of existing instruments, development of a new instrument, and cognitive interviews with adolescents to test and refine the instrument. The methods and results from each of these stages of research are presented in chronological order. Authors were additionally mindful of the criteria for reporting qualitative studies checklist suggested by Tong et al. [18].

## Concept identification research

### Methods

First, a focused review of literature was conducted to identify concepts for measuring functional outcomes that are relevant to adolescents with ADHD. Articles published between January 2001 and July 2011 that included terms for ADHD, functional outcomes and patient-reported endpoints were reviewed. Concepts were identified through the review, and were also elicited from clinical and regulatory guidelines on ADHD, selected conference abstracts, labels of medicines approved for the treatment of ADHD and results from market research with ADHD adolescents. The review was conducted in 2011 and methods and results have been described elsewhere [19, 20].

An interview guide was developed from the review of the literature. Clinicians and teachers were interviewed via telephone to identify any additional concepts related to functional outcomes that are relevant to adolescents with ADHD. Clinicians were asked about goals of therapy, treatment outcomes in terms of patient views, patient characteristics that may influence outcomes and response to pharmacological therapies, and whether other clinical aspects should be considered when evaluating benefits of ADHD medication. Teachers were asked how symptoms of ADHD manifest in a school setting, the observed impact on functional impairments during school, ways in which adolescents cope with ADHD, perception of medication efficacy and experience with existing questionnaires.

### Results

Interviews were conducted with two teachers from public schools and one from a private school, four psychiatrists, one family medicine doctor, one behavioural neurologist and three psychologists. Interviews with teachers identified the impacts of ADHD in the classroom and on school work. For example, difficulty settling in, disrupting the classroom, not following instructions, impact on academic performance, and being disrespectful or argumentative. Interviews with clinicians highlighted broader concepts that were important to understanding the impact of ADHD on adolescents: needing longer duration of symptom control to be able to do activities in the evening like homework or differences in manifestation of symptoms in adolescents (i.e., more inner-restlessness – fidgeting rather than hyperactivity per se).

The interviews also helped to identify points to consider for collecting information about functional outcomes from adolescents. For instance, some adolescents may lack insight into the impact of ADHD; adolescents’ and primary caregivers’ perspectives of impact may differ; clinicians also cautioned that adolescent perceptions/recall are often coloured by their mood at the time of reporting [19]. Clinicians also helped to focus the investigation on concepts that were clinically relevant and likely to change as a result of medical treatments. For example, clinicians suggested that the impact of ADHD on social interactions may be more relevant to evaluate than the more general concept of ‘social function’; the latter may not improve with medical treatment alone, as other variables like improvement in social skills, changes to peer attitudes and adolescents’ self-confidence may also need to change to result in improvement in social functioning.

Our research indicated that there was a paucity of literature on adolescents’ self-report of experience with the impacts of ADHD on day-to-day functioning. A qualitative study was necessary to obtain adolescents’ unique perspectives and elicit concepts regarding the impact of ADHD on their functioning.

The literature review and clinician interviews also highlighted the need for a study design for concept elicitation that would be sensitive to the potential for positive illusory bias (PIB) (which is an overestimation of *one’s own competence* compared with a criterion of competence [21]) in this population. Hence, although concept elicitation interviews with only the adolescents in the absence of caregivers are recommended to inform the development of Pediatric PRO Instruments for Research to Support Medical Product Labeling [22], our research suggested the need to also conduct interviews with caregivers to ensure that concept elicitation captured all the impacts of ADHD on functioning. A special interviewing strategy was developed to address these challenges. The interview strategy is described in the section describing the methods of concept elicitation interviews.

The results of the concept identification research were also used to develop a preliminary framework to assess the impact of ADHD on adolescents functioning. The resulting framework was used to probe on aspects of the adolescent experience with ADHD that had the greatest impact on their functioning, in the context of evaluating outcomes of pharmacological interventions for ADHD. This framework included concepts on the impact of ADHD on every day activities, social interactions and emotional function.

## Concept elicitation research

### Methods

Concept elicitation interviews were conducted with adolescents diagnosed with ADHD and their primary caregivers. The study received institutional review board approval (Schulman Associates IRB, Inc. Cincinnati, Ohio; 27 Feb 2012 a) and all participants provided written consent prior to participating in the studies (adolescents provided assent; their caregivers provided consent).

Subjects were recruited from seven clinical sites from different regions in the USA. A purposive sampling method, usually used for concept elicitation and cognitive interview studies [23, 24] was used to recruit the sample. To be eligible for participation, adolescents (aged 13–17 years at the time of consent) were required to have a clinician-confirmed (indicated by chart review) diagnosis of ADHD and no other Axis I or II, neurological or psychiatric conditions as per The Diagnostic and Statistical Manual of Mental Disorders, Fourth Edition, Text Revision; been either off medication for at least 1 week (if on stimulants) or at least 4 weeks (if on non-stimulants), or on a stable dose of medication for at least the previous 4 weeks. Additionally, adolescents must have been able to function at an age-appropriate level intellectually, as deemed by the study investigator, barring adolescents with any neuropsychological issues. All primary caregivers (at least 18 years of age) must have lived with the recruited ADHD adolescent and been the primary caregiver for the adolescent for at least the previous 6 months. Both adolescents and caregivers were not eligible to participate if they were currently enrolled in a clinical trial for an ADHD treatment or had financial affiliations with the clinical recruiting site. All interviews were conducted in English and audio-recorded so that transcripts could be prepared, reviewed and analysed. The conceptual framework from the concept identification research was used to inform the structure and content of the concept elicitation interview guide. Concept elicitation interviews started with an open-ended discussion about the impact of ADHD on functioning, followed by questions about everyday activities (using the reconstruction of a typical school day, to ensure that the impacts of ADHD at different times of the day were explored), social interactions and emotional functioning. At the end of the interview, other concepts such as impact on non-school days, weekends or holiday activities were discussed. All interviews were conducted in person and each interview took approximately 90 min to complete.

The data collection approach for the study was designed to elicit concepts to avoid missing insights about the impact of ADHD on the adolescent as a result of PIB. Data were collected from two cohorts. Cohort 1 involved separate one-to-one interviews that enabled concept elicitation from the adolescent without the caregiver and from the caregiver without the adolescent.

For Cohort 2, adolescents and caregivers were interviewed together. During Cohort 2 interviews, questions were addressed to the adolescent first and then to the caregiver for further contributions to the discussion. Caregivers were also encouraged to remind their adolescent about specific examples if the adolescent had not mentioned them. At the end of the interview, adolescents were interviewed on their own to provide an opportunity to discuss impacts that they may have hesitated to share in the presence of their caregiver.

Adolescents and caregivers also completed a set of self-administered questionnaires, including a socio-demographic questionnaire at the end of the interviews to help characterize the sample. All subjects were compensated for the time spent for participation in the study.

A content analysis approach was used to analyse data from the interviewers’ field notes, and from the transcripts of audio-recorded interviews. A phenomenological approach [25] was used to identify concepts that were relevant to explore adolescents’ experiences with ADHD. A coding dictionary was developed based on the themes and concepts that emerged during the discussions. Words and phrases provided by the subjects were selected using the coding dictionary and grouped into key themes, attributes, concepts and relationships. Analysis of the content of coded outputs was conducted by two of the authors (RP and AH); any disagreements were discussed with a third author (JS). Content analysis was an iterative process, involving review and discussion between the researchers. All agreements were based on consensus.

Concepts were reviewed on a concept tracking grid to ensure saturation (i.e., that emerging concepts adequately reflected all aspects of a measurement concept from the perspective of the patient population of interest). The ATLAS.ti program version 7.0 was used to organize the qualitative data for analysis. The outputs of the analyses were used to inform item generation.

### Results

#### Sample

Of the 60 adolescents who participated in the concept elicitation interviews, 67.0 % were male, 85.0 % were white, and 78.0 % lived in families with both primary caregivers. All adolescent participants were full-time students in the 7th–12th grade. Most adolescents were currently taking medication (83.3 %), and described the severity of their ADHD as mild (60.0 %). Interestingly, only 13.0 % reported that they were responsible for managing their medication without a caregiver’s aid.

The primary caregivers interviewed included mothers (88.3 %), fathers (8.3 %) and a grandparent (3.3 %); 50.0 % worked full-time and 13.3 % self-reported that they had also been diagnosed with ADHD.

Sample demographics and clinical characteristics from the concept elicitation and cognitive interview studies are shown in Table 1.

**Table 1:** Sociodemographic and clinical characteristics of adolescents with ADHD

	Concept elicitation study			Cognitive interview study		
	Young ADHD adolescents, 13–14 years (n = 25)	Older ADHD adolescents, 15–17 years (n = 35)	Total sample (N = 60)	Young ADHD adolescents, 13–14 years (n = 13)	Older ADHD adolescents, 15–17 years (n = 12)	Total sample (N = 25)
Sociodemographic characteristics						
Mean (SD) age, years	13.8 (0.6)	16.2 (0.8)	15.25 (1.4)	13.6 (0.5)	16.0 (0.7)	14.8 (1.4)
Male, n (%)	20 (80.0)	20 (57.1)	40 (66.7)	11 (84.6)	7 (58.3)	18 (72.0)
Racial background*^,†,‡,§,¶^, n (%)						
White	22 (88.0)	29 (82.9)	51 (85.0)	12 (92.3)	12 (100.0)	24 (96.0)
Hispanic or Latino	5 (20.0)	9 (25.7)	14 (23.3)	2 (15.4)	0 (0.0)	2 (8.0)
Black or African American	3 (12.0)	5 (14.3)	8 (13.3)	0 (0.0)	1 (8.3)	1 (4.0)
Asian	3 (12.0)	3 (8.6)	6 (10.0)	1 (7.7)	2 (16.7)	3 (12.0)
Other	3 (12.0)^†^	3 (8.6)^‡^	6 (10.0)	1 (7.7)^§^	3 (25.0)^¶^	4 (16.0)
Living/domestic situation, n (%)						
Live with both parents (caregivers) in the same home	20 (80.0)	27 (77.1)	47 (78.3)	13 (100.0)	10 (83.3)	23 (92.0)
Live with single parent/caregiver	3 (12.0)	5 (14.3)	8 (13.3)	0 (0.0)	2 (16.7)	2 (8%)
Live with both parents/caregivers in different homes (shared custody)	2 (8.0)	1 (2.9)	3 (5.0)			
Live with guardian		2 (5.7)	2 (3.3)			
Clinical characteristics						
Mean (SD) duration of ADHD, years**	5.33 (2.3)	6.03 (3.8)	5.74 (3.24)	NR	NR	NR
Diagnoses from clinical chart review (DSM code) [28], n (%)**						
ADHD, code not specified	3 (12.0)	5 (14.3)	8 (13.3)	0 (0.0)	0 (0.0)	0 (0.0)
ADHD, predominantly inattentive type (314.00)	6 (24.0)	14 (40.0)	20 (33.3)	6 (46.1)	10 (83.3)	16 (64.0)
ADHD, predominantly hyperactive/impulsive type or combined (314.01)	16 (64.0)^††^	16 (45.7)^‡‡^	32 (53.3)	7 (53.8)^§§^	2 (16.7)^§§^	9 (36.0)
Adolescent self-reported ADHD severity on day of interview, n (%)^¶¶^						
Mild	16 (64.0)	20 (57.1)	36 (60.0)	9 (69.2)	7 (58.3)	16 (64.0)
Moderate	7 (28.0)	14 (40.0)	21 (35.0)	3 (23.1)	4 (33.3)	7 (28.0)
Severe	2 (8.0)	1 (2.9)	3 (5.0)	1 (7.7)	1 (8.3)	2 (4.0)
Treatments for ADHD, n (%)**^,†††^						
Medications or drugs	19 (76.0)	31 (88.6)	50 (83.3)	13 (100.0)	12 (100.0)	25 (100.0)
Counselling/therapy	6 (24.0)	3 (8.6)	9 (15.0)	3 (23.1)	2 (16.7)	5 (20.0)
Coaching	1 (4.0)	2 (5.7)	3 (5.0)	1 (7.7)	0 (0.0)	1 (4.0)
Specialized assistance at school	12 (48.0)	12 (34.3)	24 (40)	5 (38.5)	2 (16.7)	7 (28.0)
Other	2 (8.0)	1 (2.9)	3 (5.0)	0 (0.0)	0 (0.0)	0 (0.0)

*Not mutually exclusive

‘Other’ includes: ^†^Kazakhstan (n = 1); Brazilian (n = 1); ^‡^Native Hawaiian or Pacific Islander (n = 1) and Asian-European (n = 1); ^§^South American (n = 1); ^¶^Native Hawaiian or Pacific Islander (n = 2) and Mediterranean (n = 1)

**From Clinician chart review; DSM- Diagnostic and Statistical Manual code

^††^Further subtypes: combined = 14; predominantly hyperactive/impulsive = 2

^‡‡^Further subtypes: combined = 13; predominantly hyperactive/impulsive = 3

^§§^Information on subtypes was not available for this sample

^¶¶^No adolescents reported their ADHD as very severe on the day of the interview

^†††^Not mutually exclusive

*NR* Not recorded, *SD* standard deviation

## Descriptions of the impact of ADHD from concept elicitation interviews

Adolescents discussed the impact of ADHD on their functioning in terms of (a) difficulties with everyday activities in various contexts such as: school (learning and extracurricular activities); home (e.g., early morning tasks, household chores, home work); leisure (after school clubs, hobbies, sports, part-time or voluntary work); and (b) difficulty interacting with: family (caregivers and siblings); friends (peer group, friends); and people in authority (e.g., teachers, coaches and team leaders). Emotional difficulties resulting from these impacts were also reported, such as feeling stressed about performing tasks and social interactions. In addition to these impacts, caregivers and a few older adolescents also reported problems with regulating emotions. Table 2 provides representative quotes for specific concepts within the three domains.

**Table 2:** Quotations from interview participants

Concept		Adolescent quotes	Caregiver quotes
Everyday activities	Morning	• I just waste a lot of time in the morning	• He goofs around a lot in the morning
		• Mom always has to be pushing me telling me like hurry up, hurry up	• I have to remind her to do her hygiene
			• She has left the house without brushing teeth
		• I would probably forget to like get dressed in the morning if it weren’t for my mother	
	School work in the classroom	• If I wasn’t paying attention in one class I would have a much harder time taking the test on the material than other students would	• The thing is that she’s distracted…
			• She will just stare at the window, stare at the wall
			• He’s not on the reading level, and the math level that he’s supposed to be on
		• When they do stuff in class I’m usually pretty slow, slower than other people	
	School work - homework	• I regret not being able to like focus….	• It’s always been difficult for her to sit down, do homework, and remember to turn it in
		• Because then I’ll get side-tracked and I’ll do something else and then I’ll run out of time, then I’ll have to do it in the morning	
	School behaviour – in the class room or during breaks	• I sort of just yell it out…	• She’s gotten into trouble with teachers by not-not keeping her mouth quiet or being respectful when she needs to be
		• I don’t remember ever getting up or why I got up	
		• I can’t like sit in one spot and just kind of like sit here like this all, like for 90 min and just go like this	
	Difficulty with leisure activities	• I’ll start playing something other than a (*musical*) piece I’m supposed to be playing	• With the focus, might get him a little distracted during a (*sports*) game when he needs to be paying attention
	Difficulty with chores and other task at home in the evenings	• Loading the dishwasher no one really likes, but it takes me apparently the longest amount of time	• He’ll say he’s going to do something like take the recyclables out back or whatever and-, you know, 2 h later or 3 h later he hasn’t
Social interactions	Difficulty interacting with family	• Usually I just go in my room and lock myself in, and just play video games and not talk to anyone	• This morning we had a big fight because as usual she’s thinking two minutes before she has to walk out the door about something she has had to do for the last couple months
		• Yelling at my sisters sometimes, being mean to my sisters-because like when I’m really stressing I’m a little edgy and I kind of snap really easily	
			• He doesn't know how to stop. It's like, if I yell, just stop, everybody will stop, but him
	Difficulty interacting with friends	• I’ll talk really fast and I won’t focus on anything-they’re talking to me, I kind of just like zone out a lot	• She also alienated herself from the rest of the class because she fell behind
	Difficulty interacting with teachers	• I’ll joke around in a teacher’s class…	• She’s been in trouble at school, uh, because, uh-you may want to talk to her about how she-with teachers and controlling her speech
		• I would start yelling at her and I got in a lot of trouble for that	
Emotional response to difficulty with ADHD	General stress	• When I have a lot of stuff after school I’ll usually be more just tensed up and just more irritated-irritable	• She’s admitted that she’s feeling more stressed, just as other things loom in her life
		• I usually get like a headache or whatever because of the stress	
	Stress related to interactions with peers, family, or authority	• Usually I’m kind of like nervous, kind of embarrassed, too, if I’m like in a large group of people at a presentation or something	• I think he’s so paralyzed by anxiety for-in social situations….
		• I get really nervous, I think, and I worry a lot and that’s how I get distracted	
	Stress related to school work and homework	• Uh, it’s just like trying to not fail. Like I really, I really hate failing, and I just hate the feeling	• She just-so she was under a lot of pressure internally obviously to get this done and just completely fell apart and, you know, was in tears and everything like that.
		• I was more worried about, oh, you know, I have to do this, I have to do this homework, I have to turn this packet in and everything, everything, everything	
			• At night if there’s too much of it she’ll have complete anxiety attacks and mental breakdowns occasionally if the pressure is just too much

When describing their difficulties, most adolescents discussed the level of effort that they needed to accomplish tasks, manage their behaviour or to engage in conversations. Adolescents provided examples to illustrate how difficult it was to do things because of their ADHD in various contexts, and shared how these difficulties stressed them out. For example, a 17-year-old girl with ADHD said: “*…this whole junior year has been very stressful, stressful…. I think it was hard, I think it’s harder for me because, ‘cause of my ADHD like, yeah, okay, my ADHD is hard to, just like very hard to focus.”*

Adolescents mainly reported difficulty with tasks that need attention. Difficulties with focus appeared to be the primary difference they notice when not on medication. They also reported difficulty with completing tasks properly and on time, remembering things and following instructions. Difficulties with behaving well during class included examples of being reprimanded by teachers for ‘blurting things out’ or not waiting their turn, and interrupting the class by moving around, fidgeting or dropping things, thus causing a distraction to others. Difficulties at home included examples of ADHD symptoms interfering with getting ready for school on time in the mornings, the need for multiple reminders from their caregivers in the mornings before school and difficulties with completing homework or chores in the evenings. They also mentioned that their ADHD symptoms had an impact on leisure activities, including the activities that they performed in after-school clubs, sports, volunteering or part-time work. Caregivers reported similar difficulties but reported impairment perceived in terms of the consistency and quality of activities performed. Caregivers also reported that the adolescent with ADHD lacked ‘motivation’ and procrastinated.

When speaking about social interactions, difficulties interacting with family members and with peers/friends were typically discussed by both adolescents and caregivers. In general, older adolescents were able to report on some specific difficulties with social interactions (e.g. difficulties with listening/not focusing on what others were saying) compared with younger adolescents. For example, a 16-year-old girl with ADHD said: “*Yeah, I’m much more-much hyper, really hyper, I’ll talk really fast and I won’t focus on anything-they’re talking to me, I kind of just like zone out a lot… Yeah, they say something and then I’m just like ‘what, repeat that, I didn’t hear you’-and they get all mad because they feel I wasn’t paying attention.”*

Older adolescents also reported on the greater demands placed on their social skills because of their ADHD. The need to listen and focus on conversations, participate in more reciprocal communication, engage in dating and group activities increased as they grew older. Parents reported seeing less of their adolescent as they grew older, and were able to provide fewer insights into the social difficulties experienced by their adolescents.

Adolescents also reported on difficulties with interacting with family members, and avoiding interactions because they were difficult. When discussing interactions with authority figures, fewer adolescents reported difficulty, although the main problem reported was regarding interactions with teachers at school. A few adolescents who participated in sports also mentioned similar problems with coaches. Caregivers also consistently reported difficulties with communication and arguments about poor performance or forgetting to do tasks. Regarding interactions with peers, a few adolescents reported problems with getting into arguments, being impulsive and lacking etiquette both in and outside of school. While adolescents reported no trouble keeping or making friends, caregivers reported adolescents were not doing as well socially as other children.

Parents typically provided additional insights into the specific difficulties that their adolescents faced as a result of other symptoms of ADHD (impulsivity and hyperactivity). For example, a caregiver of a 15-year-old boy with ADHD reported on the difficulty their child had because of being impulsive, but was also able to report on change to this difficulty over time: “*…they-these kids come to see my younger son…. um, so if they’re doing something… playing on the computer or on the trampoline. But there are, I’m sorry, there are times when, um [Son with ADHD], again, will get very upset with these neighbor kids because something-something he has perceived that they’ve done, um, and that is better though now than it used to be. A few years ago he would-he might beat them up, um, but now he just might, I don’t know, walk away or say something rude to them or something, it’s much less, um, physical than it used to be.”* Another caregiver reported: *“I think, um…I think [<Son>] has social challenges. I don’t think he thinks he does, um, I think they are definitely related to his A-ADHD, um, and that’s because he gets in your face when he’s not on medication and he’s jumping around and moving around and it’s just-it’s irritating. And I could see where his peers would get tired of that fast, where he can’t just have a conversation.”* Further examples are provided in Table 2.

Most adolescents described feelings of stress related to their performance of tasks or social interactions because of their ADHD, using terms such as guilt, worry, anxiety, stress/stressed out and irritable to describe their emotions. In the school setting, adolescents provided examples of stress with completing classroom work properly and on time, remembering things, following instructions, behaving well during class, interacting with teachers and peers. Adolescents reported feeling stressed in school owing to not understanding the work, not keeping up with the rest of their class during lessons, and getting behind in school work largely owing to their difficulty with keeping focused on lessons. Adolescents provided examples of stress, such as getting ready for the day on time in the mornings, completing homework or chores in the evenings, and interacting with family members. Parents used the term anxiety to describe the adolescents’ experiences and reported on difficulties that adolescents had with the control of their emotions, mostly anger, in terms of temper outbursts. A few caregivers also mentioned excessive sadness in the adolescent.

ADHD significantly impacts aspects of adolescent functioning. Adolescents and caregivers provided different perspectives of the adolescents’ experiences of ADHD. For example, in an interview in Cohort 1 where the adolescent and his caregiver were interviewed separately, the caregiver reported that her son with ADHD was in a tagging crew (a form of gang that is involved with activities such as illegally spraying graffiti across public property), and on occasion she has had to phone the police regarding her son to help discipline her child’s illegal activities. In the individual interview with the son, despite being probed about getting into trouble, he did not mention this.

While caregivers were more open about reporting on incidents where adolescents had experienced difficulties, with prompts adolescents did not refute these incidents. This is illustrated in the quote below, during a discussion about the impact of ADHD on leisure activities from a Cohort 2 interview, where the caregiver and adolescent were interviewed at the same time.

Parent: “….*So just because he’s doing it and liking it doesn’t mean he’s happy about it in the end*.” Interviewer: “*Why would you say that is then?*” Adolescent: “*Um, well, like sometimes when I’m playing FIFA I will keep-it will start to get boring after I play a couple of games. So then I’ll go outside and ride my bike. And so I can’t just stay on one thing for like the whole day*.”

The concept tracking grid to evaluate saturation of concepts showed that across interviews, adolescents reported similar difficulties in the context of home, school and leisure, supporting saturation.

## Develop a conceptual framework

**Fig. 1:**
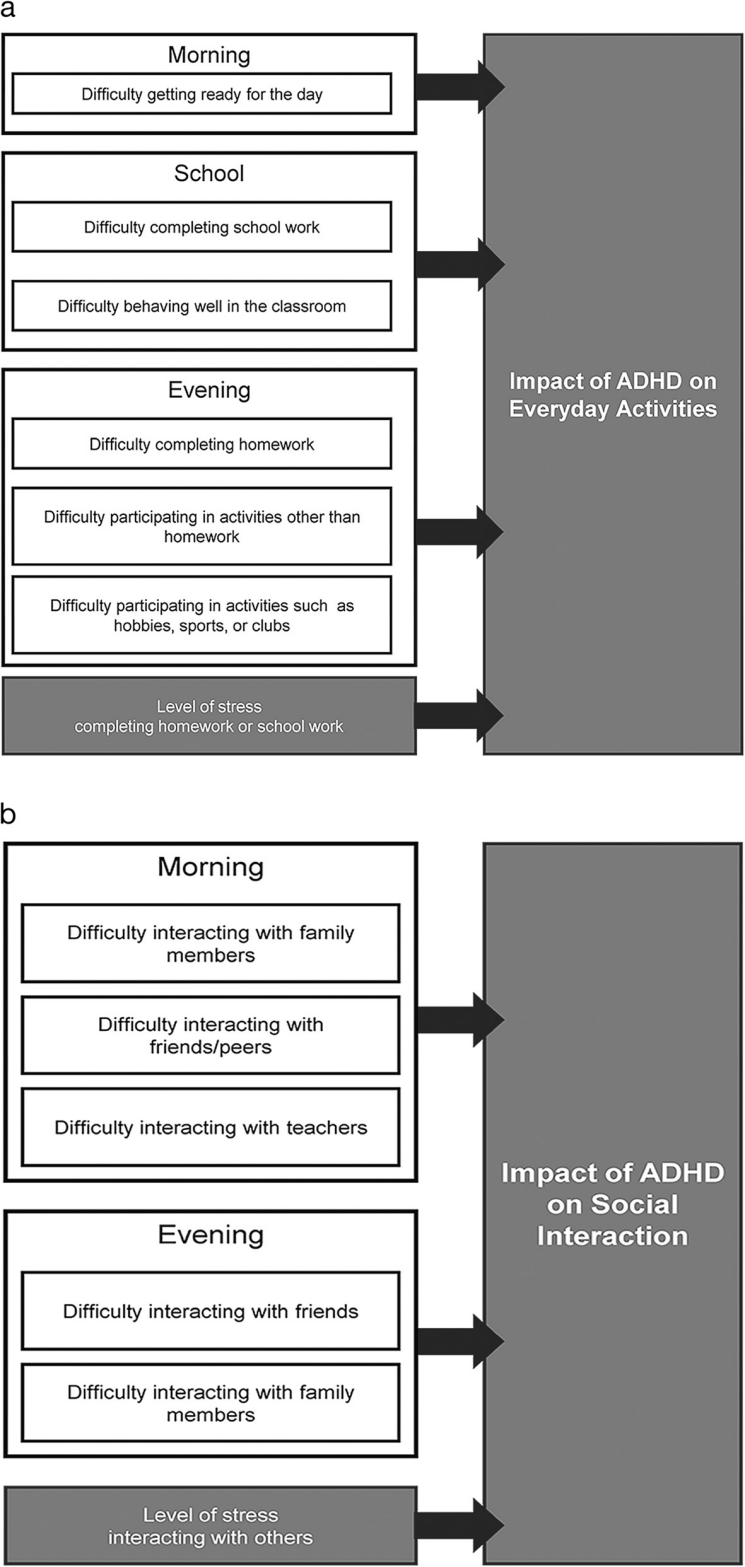
Impact of ADHD. **a** Everyday activities. **b** social interactions

The concepts identified through concept elicitation interviews were used to revise the preliminary conceptual framework developed previously based on the concept identification work. The revised framework was shared with clinicians and was modified further based on their feedback, to ensure clinical relevance and to highlight concepts that were more useful to capture the outcomes of pharmacological interventions for ADHD (the context of use for the new instrument). This resulted in a few revisions to the conceptual framework for measuring the concept of interest. For example, clinicians suggested that some of the concepts reported by adolescents and their caregiver as an impact of ADHD, such as emotional lability, were likely to be part of the psychopathology of the disease rather than an impact of the core symptoms of ADHD. Clinicians also helped to focus the concepts in the framework on the impact of the symptoms of ADHD on function, rather than the functional impairments noticed by most caregivers of adolescents without ADHD. The results also showed that social functioning in adolescents was closely linked to the social settings to which they are exposed, rather than just being an individual with ADHD. Clinician interviews had highlighted previously that social functioning may not respond to pharmacological treatments alone. Therefore, the concept to evaluate adolescents’ experiences with ADHD was focused on difficulty with social interactions, rather than more distal concepts about their social functioning (e.g., making/keeping friends, getting into the wrong groups), as these would be influenced by environmental and social factors other than ADHD. Based on the results above, the following concepts were considered as core concepts to collect data from adolescents to understand the impact of ADHD on adolescent functioning, and relevant to evaluate outcomes of pharmacological treatment. The final conceptual framework for evaluating the impact of ADHD on everyday activities and social interactions in adolescents with ADHD is illustrated in Fig. 1.

Adolescents reported that the level of difficulty varied at different times of the day. They also reported that they would find it hard to recall their difficulties more than a few days in the past.

## Tool review

### Methods

To determine whether published tools that measure functional outcomes in ADHD covered the concepts that are meaningful and important to adolescents with ADHD, a review was conducted to identify tools used in clinical trials in adolescent ADHD. PRO instruments were identified based on a review of select conference abstracts, clinical trials.gov, the Patient-Reported Outcome and Quality of Life Instruments Database (PROQOLID), and the Patient Reported Outcomes Measurement Information System (PROMIS) database. The contents of the tools were reviewed to identify a) the best coverage of concepts emerging from the concept elicitation work and b) methods used to generate the items and assess if they meet criteria for content validity required for PRO tools used to support label claims [16, 17]. This produced a short list of tools for further examination of the domains and specific items used to evaluate functional outcomes for their relevance for the adolescent ADHD population.

### Results

Thirty-four tools were identified from the review; 11 had adolescent-specific versions. An item ‘pool’ was developed, which included the items selected from instruments that cover the concepts from the conceptual framework. Adolescent-specific versions largely included concepts similar to the child versions, with some adjustments to match developmental stage, and were more likely to be self-reported.

In reviewing the content of the tools, the Weiss Functional Impairment Rating Scale-Self (WFIRS-S), a self-reported scale to evaluate symptoms and functioning in adults with ADHD, covers seven conceptual domains (family, work, school, life skills, self-concepts, social and risk), and had the best coverage of concepts. However, the tool has some shortcomings in terms of suitability of use for evaluating outcomes of pharmacological interventions from the adolescent’s perspective. Firstly, item generation for the tool was not supported by concept elicitation work with an adolescent population. Additionally, the recall period of the tool is the ‘past month’, which may not capture the variability of the impact of symptoms on functioning as experienced by varying demands of the days for adolescents and would be challenging for adolescents to complete as they mentioned difficulty recalling impacts over longer periods of time. Finally, the WFIRS-S collects information about the frequency of impacts rather than the level of difficulty of functioning experienced and reported by the adolescent with ADHD. Based on the review, development of a new instrument was deemed the most appropriate strategy.

### Item generation

The findings from concept elicitation interviews and results of instrument review suggested the need for an instrument: a) with a shorter recall period; b) that collected information about the level of difficulty and effort required by the adolescents; and c) that evaluates the impact of ADHD on functioning in different contexts (school, home, and leisure) from the adolescent’s perspective. The tool review found no such instrument to fill this need.

Best practice guidelines were used to guide the process of generating items [17]. This included the creation of questions, recall period and response choices. Items were generated using patient terminology to reflect the concepts relevant to adolescents’ understanding of their experience of impacts from ADHD as defined in the modified conceptual framework. A pool of items identified from existing questionnaires during concept identification was used as a reference to inform item construction. For formulation of the questions and the construction of the instrument, methods outlined by Streiner and Norman [26], were followed. Item generation started with an all-day meeting where the results from the qualitative concept elicitation interviews, clinician feedback, and considerations from the initial concept identification research were discussed. Input from the instrument development team members (authors of this manuscript) were used to construct the items. Items were developed using an iterative process of: item development, review and revisions. Review and discussions referred back to qualitative data from the interviews to inform the decision-making process. External reviews of the instrument were conducted by ADHD clinicians and translatability experts.

Concept elicitation research suggested that the impact of ADHD on functioning was reported by adolescents in terms of the *level of difficulty or effort* required to do their school work, leisure activities and tasks at home. Response options for the instrument were thus selected to enable adolescents to specify how hard it was to do/how much effort was required, to function. Two types of scales for obtaining response options – a numeric rating scale (NRS) and a visual analogue scale (VAS) – were developed for testing in cognitive interviews.

To capture the variability of the adolescent experience over the course of a day, and to ensure shorter recall periods to address recall bias, the instrument was designed to be administered twice daily (at the end of the school day and in the evening before bed). An electronic platform was selected to facilitate the ease of completion, enhance compliance (with the inclusion of features such as the ability to set time windows and reminder alarms to facilitate the completion of the instrument during the specified timeframes), include examples of terms on the screen, and also ‘pop-up’ definitions if the participant requires additional information. At the end of the school day, adolescents were asked to indicate the magnitude of difficulty or effort required to do the following – ‘how hard was it to’: get ready for the day in the morning, do school work, behave well in the classroom, interact with family members in the morning and/or interact with friends and teachers at school. Another item asked how stressed they felt about school work that day (seven items). At the end of the day, adolescents were asked to indicate the magnitude of difficulty or effort required to do the following – ‘how hard was it to’: do homework, chores, sports or hobbies and interact with friends and family. Another item asked how stressed they felt about doing their homework that evening (six items).

**Fig. 2:**
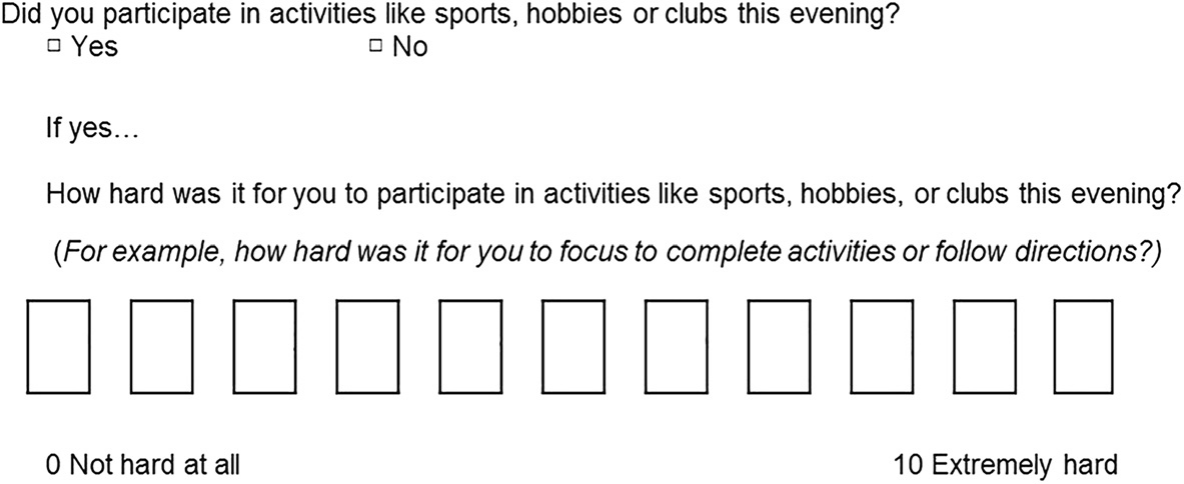
Sample item from instrument

The instrument also includes items that ask the adolescent to indicate their overall level of stress at three time points in the day – morning, school day and evening, and some optional items that can help to characterize the adolescents’ experience such as: “Did you leave home on time this morning?” and “Did you have all the materials that you needed to complete your homework or other work for school?”. Figure 2 shows an example question from the instrument. The final conceptual framework and scoring methods will be determined following psychometric validation studies.

A translatability assessment and a lexibility (reading level) evaluation were conducted on the draft instrument prior to testing the instrument to ensure that the items were translatable and that the reading level was appropriate for adolescents aged 13–17 years. The instrument was finalized for cognitive testing. The translatability assessment helped to identify words or phrases that were structurally or culturally problematic when translated into different languages for potential use in multicountry clinical trials. This included revisions to the diary such as spelling out times of the day as an alternative to a 24-hour clock, as well as rephrasing statements and replacing words for clarity such as ‘to reach transportation for school’ instead of ‘to get transportation to school’.

## Cognitive interviews

### Method

Cognitive interviews were conducted to gain insight into subjects’ interpretations of the items, match the intent of the items and assess whether any critical content (concepts or items) had been omitted from the instrument. All interviews also explored the suitability and appropriateness of response options and recall periods, and also preferences for an NRS or a VAS to select responses to items [17]. The patient population was recruited using similar criteria and narrow age groupings from the concept elicitation study. Two rounds of interviews were conducted to allow for adjustments. Round 1 included eight adolescents and Round 2 included 17 adolescent interviews. Institutional review board approval (Schulman Associates IRB, Inc. Cincinnati, Ohio; 16 May 2013) was received and all participants provided written consent prior to participating in the study (provided assent and consent as required). All interviews were conducted in English and audio-recorded so that transcripts could be prepared, reviewed and analysed.

Feedback received from adolescents with ADHD during the cognitive interviews was used to improve the questionnaire. The analyses of data from these interviews included examining: 1) the clarity of the items within the scale; 2) participants’ interpretation of the items; 3) ease of questionnaire completion; 4) comprehensiveness and relevance of the measure; and 5) appropriateness of the format, response scales and recall period. The analysis also focused on the instructions and content coverage of the questionnaires to ensure items fully captured the impact of ADHD on functioning. After the completion of Round 1 (n = 8 interviews), an interim analysis was conducted using the criteria listed, and modifications were made accordingly and retested during Round 2 interviews (n = 17 interviews) before finalizing the instrument.

### Results

Twenty-five adolescents were interviewed across the two rounds of interviews. Similar to the sample for concept elicitation interviews, all of the adolescents included in the cognitive interview study were currently taking medication (100 %) and most described their ADHD as mild (64 %). A smaller percentage of adolescents in the cognitive interview study (28 %) reported having specialized assistance at school compared with those included in the concept elicitation study (40 %) (Table 1).

All adolescents were able to complete the instrument. Specifically, adolescents reported that the instructions were clear, the content of each question was appropriate, and that they understood the content of the questions as it was intended by the instrument developer. None of the adolescents mentioned any concepts that were missing. A range of responses was chosen by adolescents, suggesting the presence on impacts of ADHD on the day of the interview. Furthermore, adolescents were able to explain why they chose a specific response option and provide an example of a time they may have chosen a different option. This helped to confirm that the instrument was well understood and that adolescents had insights into the impact of ADHD on their functioning. Results also showed that for response options, an NRS was preferred over the VAS. Overall, all the changes made to the instrument were minor. These changes included switching the order of text, removing unnecessary text and/or adding in words or phrases to help to clarify the intent of the items. A term change for emotional functioning that emerged was to change ‘stressed’ to ‘worried’ for interactions with others in the evening. In addition, modifications were made to the ‘pop-up’ descriptions developed to facilitate adolescents’ understanding of items. These statements or descriptions usually included examples of activities associated with the item such as ‘difficulty focusing while talking, family members not understanding’, and were therefore modified to be more relevant to the adolescents’ experiences. One participant (16-year-old boy) suggested changing the name from diary. *“…I would I don’t know, maybe-I’d just say test or complete this packet. Just not diary, no, with that I think of like a 12- to 14-year-old girl writing about who knows what.”* The name of the instrument was hence revised to ‘a schedule’ instead of ‘diary’. The results of this research confirmed the impact of ADHD on adolescent functioning that is mentioned in the literature, and highlights concepts that should be evaluated to understand treatment outcomes that are meaningful to adolescents.

## Discussion

Most children with ADHD continue to suffer impacts associated with the disease into adolescence, despite receiving treatment. This has been attributed to the increasing demands placed on adolescents at school and at home [5, 6]. While this has been reported through case series, little is known about the full scope of the impact of ADHD on adolescents’ function. Additionally, most instruments developed to evaluate outcomes of treatment in this population rely on parent or physician reports rather than direct reports from adolescents [11], which may impact our understanding of outcomes that are most important to adolescents. This study aimed to address these gaps by first identifying and defining the concepts that have the greatest impact of ADHD on adolescents’ functioning, from the perspective of the adolescent and secondly, using the results to inform the development of a new instrument.

### Methods for identifying and defining the concepts from adolescents with ADHD

Concepts related to the impacts of ADHD and methodological considerations identified from concept identification phase were used to design the study and informed the data collection approach. As low concordance between reports by parents and adolescents was reported in the literature [13], the qualitative research study was designed to supplement data collected from interviews with adolescents, with caregiver interviews. The design also explored options for data to be collected separately from both perspectives and together. This approach enabled cross-examination of both perspectives, addressing potential PIB in conditions where deficits in executive functioning may have been present, as was reported by Owens et al. [21]. Additionally, taking into consideration attention deficit and potential difficulty with recalling experiences, and based on feedback from clinicians, a predefined structure using a day reconstruction approach, as well as use of specific probes, facilitated concept elicitation. Insights gathered from both adolescents and caregivers provide a more comprehensive picture in describing the impact of ADHD on adolescent functioning, suggesting the value of conducting concept elicitation interviews with both adolescents and their caregivers. Adolescents with ADHD reported more about the difficulties and the degree of effort that they required to perform their day-to-day activities than parents who reported on the occurrence and frequency of the impacts that they observed. While the findings of this study supports the value in capturing the perspectives from caregivers and adolescents, further study is required to explore the pros and cons of interviewing adolescents and their caregiver separately.

The qualitative study provided evidence supporting adolescents’ ability to self-report on the impact of ADHD. Concept elicitation interviews identified functional concepts of impairment including: difficulty performing tasks; difficulty related to interactions; and feelings of stress about difficulty with school work and performing tasks. The findings are similar to Goodman et al. [6], with respect to reports of greater demands in adolescents in functional areas such as academic and social settings. While the adolescents provided insight to their academic and social settings, the caregivers were able to report supplemental details about observations from their perspectives. The caregivers, for example, could comment on the number of social activities while the adolescents could comment about how ADHD symptoms impaired their social interactions. The dynamic between adolescent experience and caregiver observation that was reported in this research is consistent with Achenbach et al. [13]. Adolescents reported the impact of ADHD on daily functioning in various contexts – at school, at home and during leisure time.

### Development and testing of a new instrument

Review of the instruments developed previously demonstrated incomplete coverage of the important concepts for assessing the impact of ADHD on functioning identified during the concept identification and concept elicitation studies. Therefore, a novel instrument was developed for adolescent self-report, focusing on outcomes that are likely to respond to successful medical intervention in the context of a clinical trial. Inclusion of clinicians’ perspective helped to ensure the clinical relevance of the tool. For example, collecting data on areas where adolescents may be able to perceive improvement (e.g., difficulty with social interaction), rather than areas where improvements are the consequence of external factors (e.g., social functioning – peers need to see the difference, then their minds need to be changed). The development of this new tool followed the processes as outlined by a USA regulatory agency [16], as well as specific development guidelines for content validity [17] and recommendations for the assessment of children and adolescents [22].

The generation of new items for the PRO instrument captures adolescents’ experiences during different times of the day. The daily variability in adolescents’ schedule, coupled with the variation of experiences and difficulties owing to ADHD, as well as the adolescents’ inherent difficulty with remembering past experiences, suggests that data regarding these concepts may need to be captured more frequently to support the need for adolescents to respond to questions based on a shorter timeframe. None of the existing PRO tools include a short recall period (e.g., WFIRS-S uses the past month recall). A short recall period was used to prevent reporter bias based on clinician report and research suggesting that ADHD impairs working memory [27].

For adolescents with ADHD the magnitude of difficulty with performing the task and the effort to perform the task appeared to be more relevant than the actual occurrence or absence of the functional impairment. Understanding the benefit of treatment in terms of whether or not it reduces the level of effort to perform tasks may resonate better with adolescents, when evaluating outcomes of treatment in clinical trials or monitoring outcomes in clinical practice. The response options that reflect changes in the level of difficulty or effort required by the adolescents if their ADHD symptoms were controlled assess two key measurement aspects of ADHD functioning. This includes the focus on the performance of daily activities rather than the perception of their performance, removing any bias in adolescents’ ability to complete a task per FDA guidance. Secondly, the focus on capturing the level of difficulty required to complete a task, rather than frequency of impairment, has the potential to provide more meaningful measurement of behaviour and functioning.

The content validity of the instrument was established through cognitive interviews where adolescents completed the instrument, providing insights on the selection and meaning of their responses. Results from these interviews suggest that adolescents had valuable insights into the impact of ADHD, and could report on these impacts, contradicting previous opinion that PIB may inhibit ADHD adolescents’ ability to self report [21]. The results from the cognitive interviews were then used to refine the new PRO instrument.

The new instrument can be used to capture the adolescent perspective of the impact of ADHD on functioning. It can also supplement traditional clinical assessments used to monitor adolescents’ experiences in clinical setting, as well as in clinical studies, hence allowing evaluation of outcomes that are meaningful to adolescents. However, there are a few limitations to the research that should be considered when evaluating the application of the instrument. To start, the sample of adolescents interviewed was relatively homogenous, with white males making up a large majority of those adolescents interviewed in the study. Most of the participants were recruited from only two states (Virginia and California) within the USA, which may not reflect the general population of ADHD participants. Additional research on the concept elicitation approach to compare concept elicitation with and without caregivers, and in different countries, ethnicities and cultures is warranted. The diagnosis and sub-types of ADHD (Table 1) were based only on information available on patients’ charts; no clinical assessments were conducted for the study to confirm the diagnosis or ADHD sub-type. Further quantitative evaluation of the instrument should also be conducted to determine methods for scoring and to explore the psychometric properties of the instrument, as well as to assess the sensitivity to change of these scores resulting from interventions.

## Conclusion

This study provides a comprehensive understanding of the impacts of ADHD on adolescent functioning, which has been used to inform the development of a new instrument for measuring outcomes. Adolescents were able to discuss the impact of ADHD on their lives in concept elicitation interviews and report the impacts of ADHD on a self-report instrument. A new instrument, that was developed based on these findings, can be used to supplement assessments in clinic settings and research, and to evaluate outcomes that are meaningful to adolescents. Scientific advocacy for the use of such instruments can be valuable to measure outcomes that are meaningful to the ADHD adolescents and the clinical community. To continue to build on this research foundation, additional quantitative work should be done to validate the concepts and instrument to facilitate improved measurement of treatment effects among adolescents.

## Abbreviations

ADHD: Attention-deficit/hyperactivity disorder
FDA: US Food and Drug Administration
NRS: Numeric rating scale
PIB: Positive illusory bias
PRO: Patient-Reported Outcome
PROMIS: Patient Reported Outcomes Measurement Information System
PROQOLID: Patient-Reported Outcome and Quality of Life Instruments Database
VAS: Visual analogue scale
WFIRS-S: Weiss Functional Impairment Rating Scale–Self
